# Genome-Wide Variants Associated With Longitudinal Survival Outcomes Among Individuals With Coronary Artery Disease

**DOI:** 10.3389/fgene.2021.661497

**Published:** 2021-06-01

**Authors:** Jennifer R. Dungan, Xue Qin, Melissa Hurdle, Carol S. Haynes, Elizabeth R. Hauser, William E. Kraus

**Affiliations:** ^1^Division of Healthcare in Adult Populations, School of Nursing, Duke University, Durham, NC, United States; ^2^School of Medicine, Duke Molecular Physiology Institute, Duke University, Durham, NC, United States; ^3^Department of Biostatistics and Bioinformatics, Duke University School of Medicine, Durham, NC, United States; ^4^Cooperative Studies Program Epidemiology Center, Durham VA Medical Center, Durham, NC, United States; ^5^Division of Cardiology, Department of Medicine, School of Medicine, Duke University, Durham, NC, United States

**Keywords:** coronary artery disease, survival analysis, genome-wide association study, age-related disease, candidate gene analyses

## Abstract

**Objective:**

Coronary artery disease (CAD) is an age-associated condition that greatly increases the risk of mortality. The purpose of this study was to identify gene variants associated with all-cause mortality among individuals with clinically phenotyped CAD using a genome-wide screening approach.

**Approach and Results:**

We performed discovery (*n* = 684), replication (*n* = 1,088), and meta-analyses (*N* = 1,503) for association of genomic variants with survival outcome using secondary data from White participants with CAD from two GWAS sub-studies of the Duke Catheterization Genetics Biorepository. We modeled time from catheterization to death or last follow-up (median 7.1 years, max 12 years) using Cox multivariable regression analysis. Target statistical screening thresholds were *p* × 10^–8^ for the discovery phase and Bonferroni-calculated *p*-values for the replication (*p* < 5.3 × 10^–4^) and meta-analysis (*p* < 1.6 × 10^–3^) phases. Genome-wide analysis of 785,945 autosomal SNPs revealed two SNPs (rs13007553 and rs587936) that had the same direction of effect across all three phases of the analysis, with suggestive *p*-value association in discovery and replication and significant meta-analysis association in models adjusted for clinical covariates. The rs13007553 SNP variant, *LINC01250*, which resides between *MYTIL* and *EIPR1*, conferred increased risk for all-cause mortality even after controlling for clinical covariates [HR 1.47, 95% CI 1.17–1.86, *p(adj)* = 1.07 × 10^–3^ (discovery), *p(adj)* = 0.03 (replication), *p(adj)* = 9.53 × 10^–5^ (meta-analysis)]. *MYT1L* is involved in neuronal differentiation. *TSSC1* is involved in endosomal recycling and is implicated in breast cancer. The rs587936 variant annotated to *DAB2IP* was associated with increased survival time [HR 0.65, 95% CI 0.51–0.83, *p(adj)* = 4.79 × 10^–4^ (discovery), *p(adj)* = 0.02 (replication), *p(adj)* = 2.25 × 10^–5^ (meta-analysis)]. *DAB2IP* is a ras/GAP tumor suppressor gene which is highly expressed in vascular tissue. *DAB2IP* has multiple lines of evidence for protection against atherosclerosis.

**Conclusion:**

Replicated findings identified two candidate genes for further study regarding association with survival in high-risk CAD patients: novel loci *LINC01250* (rs13007553) and biologically relevant candidate *DAB2IP* (rs587936). These candidates did not overlap with validated longevity candidate genes. Future research could further define the role of common variants in survival outcomes for people with CAD and, ultimately, improve longitudinal outcomes for these patients.

## Introduction

Worldwide, more people die from heart disease than from any other cause (Virani et al., 2020). Up to half of the variation in CAD etiology has been attributed to genetic influences. In a 36-year observational study of nearly 21,000 Swedish individuals who were twins, researchers estimated the heritability of risk of death from CAD to be 38–57% (Zdravkovic et al., 2002). Investigators in a Danish twin study achieved similar results, estimating the heritability of CHD mortality at 55% even after controlling for smoking and BMI (Wienke et al., 2005). Taken together, these results suggest that genetic factors are significant contributors to the risk of death from CAD.

Researchers have tested a limited number of CAD candidate genes for associations with mortality endpoints, with inconsistent findings. Candidates such as *ADAM33, AGT, AGE*, and *ILRL1* have only been tested with short-term endpoints, showing variable effects and remaining unreplicated (Gioli-Pereira et al., 2012; Ellis et al., 2013; Figarska et al., 2013; Patel et al., 2019). Variants in the well-studied 9p21 and 6p24 CAD candidate loci have also demonstrated sex- and age-associated effects on risk of cardiac death (Ivanova et al., 2017). Previously, we identified a genetic component of survival in the context of clinically significant CAD—a novel phenotype we characterized as “survivorship with CAD” (Dungan et al., 2013). In that study, we conceptualized the genetic contribution to survivorship with CAD as age and phenotype dependent and as likely to share genetic variation with the lifespan longevity phenotype. Using this framework, we tested known CAD candidate genes for association with survival outcomes specifically in patients with prevalent CAD. We identified SNPs in *LSAMP* that had varied allelic effects on hazards of all-cause mortality, with some alleles conferring significantly increased hazard of all-cause mortality and others significantly improved likelihood of survival. Notably, these observed allelic effects were specific to CAD cases, having shown no significant association among non-CAD controls (Dungan et al., 2016). Following up on our prior candidate-gene results with an agnostic, genome-wide association analysis is an appropriate next step in the search for genomic variation that contributes to long-term survival outcomes among people with CAD.

The purpose of this study, then, was to perform a genome-wide association screen for variants associated with survival among people with CAD to inform future hypothesis-driven work. We include replication and meta-analyses.

## Materials and Methods

### Design

We conducted a secondary analysis of existing data from two separate GWAS sub-studies of participants sampled from the Catheterization Genetics study clinical cardiovascular biorepository (CATHGEN; *N* = 9,334; Sutton et al., 2008; Kraus et al., 2015a, b). We employed a two-step genome-wide association screen for variants associated with survival outcomes in patients with CAD, using the separate GWAS sub-studies for discovery (*N* = 684) and replication (*N* = 404).

### Study Population

**FIGURE 1:**
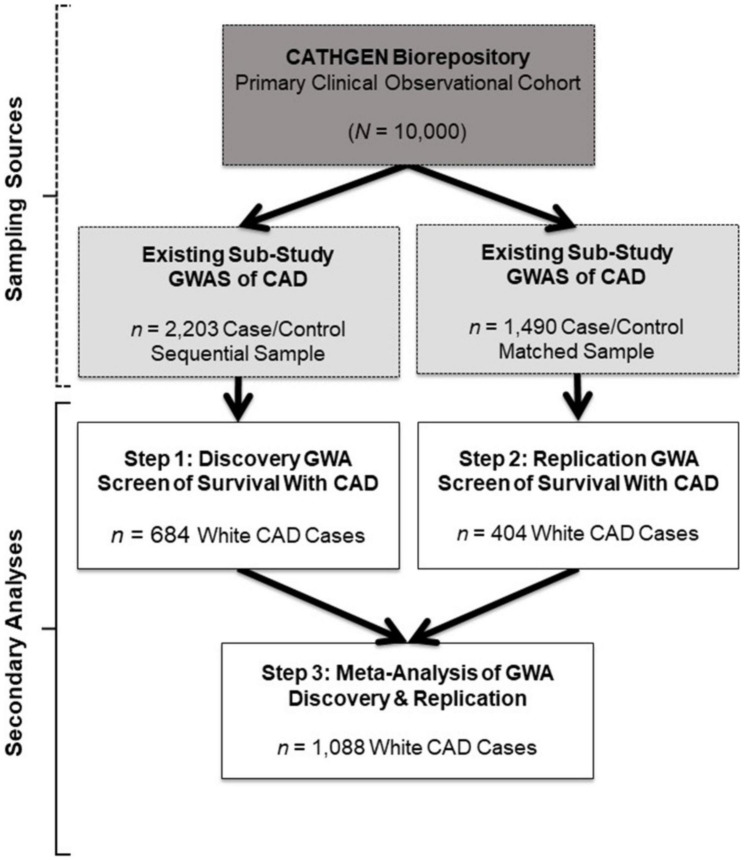
Study design and sample schema. This figure depicts the primary study (dark gray box) and extant sub-study data (light gray boxes) from which our retrospective datasets for the present analyses were derived (white boxes). The CATHGEN Biorepository containing data from 10,000 individuals recruited after cardiac catheterization (dark gray box) supplied the samples for two separate GWASs of CAD sub-studies (light gray boxes), providing the extant GWAS genotype data for our secondary analysis. The first GWAS sub-study contained data from a sequential sample of 2,203 CATHGEN CAD cases and controls (light gray box, left); the other CAD GWAS sub-study had data from 1,490 CATHGEN CAD cases and matched controls (light gray box, right). The white boxes display the secondary datasets we analyzed in the present retrospective study. Specifically, we derived our discovery cohort of 684 White CAD cases (white box, left) from the sequential case-control GWAS data; this discovery dataset was analyzed for Step 1. Our replication cohort of 404 White CAD cases (white box, right) was derived from the matched case-control GWAS dataset; this replication dataset was analyzed for Step 2. We then performed meta-analyses of our discovery and replication cohorts (black arrows converging on the bottom white box) for Step 3.

The Duke University Institutional Review Board approved the primary CATHGEN cohort biorepository, GWAS sub-studies and the present GWA screen of survival in CAD. Briefly, patients were referred to CATHGEN from cardiac catheterization laboratories at Duke University (southeastern United States), where they were being evaluated for ischemic heart disease. Patients with severe pulmonary hypertension or transplant were ineligible for CATHGEN participation. All participants provided informed, written consent for participation in CATHGEN at the time of enrollment, which included DNA collection, medical record abstraction, and annual follow-up for mortality events. CATHGEN primary investigators drew samples from the primary CATHGEN cohort biorepository for the two GWAS sub-studies we evaluated in the present secondary analysis (gray boxes, Figure 1). The first sample was a sequential series of 2,023 CATHGEN participants > 18 years of age whose primary reason for catheterization was concern for ischemic heart disease with complete coronary angiogram (Figure 1, gray box, left; Kraus et al., 2015b). The other sub-study sample (*N* = 1,490) consisted of a CAD case–control set of the same age and clinical indication, matched to controls on age, race, and sex (745 CAD cases and 745 non-CAD controls; Figure 1 gray box, right; Kraus et al., 2015b).

#### Inclusion Criteria

We further applied the following criteria to the existing GWAS sub-study cohorts for inclusion in the present study’s analyses. We included only self-reported White participants for two reasons: First, the frequency of self-reported Black/African American individuals with CAD in the extant GWAS datasets was insufficient for stratified analyses (*n* = 126 and 124 for the discovery and replication cohorts, respectively). Second, our goal is for the present GWA screen to be comparable to the findings of our prior work as we look to build genomic convergence for this phenotype, and the sample for our earlier candidate gene study of survivorship with CAD comprised White individuals (Dungan et al., 2016).

We selected CAD-defined cases from the larger of the two sub-studies to serve as our GWAS discovery dataset (*n* = 684). As in the primary CATHGEN study, we defined positive CAD case status as having a Duke CAD index ≥ 32 (at least one vessel having at least 75% stenosis) determined by clinical coronary heart catheterization (Sutton et al., 2008). Of note, the Duke CAD index reflects both the extent and location of stenosis. It is used as an indicator of disease severity and includes individuals with left main coronary disease. Likewise, we selected CAD cases from the smaller GWAS sub-study to serve as the replication dataset (*n* = 404).

#### Exclusion Criteria

As we were specifically interested in survival outcomes among people with CAD, we excluded the non-CAD controls from our analysis. We excluded subjects for whom valvular heart disease was either the primary or secondary indication for coronary catheterization and those who had pulmonary hypertension, transplant, right heart catheterization, congenital heart disease, severe congestive heart failure, or peripheral arterial disease intervention. We excluded participants from analyses if they died within 14 days of their initial catheterization in order to mitigate any undue influence of mortality due to procedural intervention on the time-to-event results.

### Data Sources, Variables, and Outcomes

We used de-identified data for our analyses. We determined survival event data as number of days from study enrollment (baseline) to all-cause mortality (event) or last follow-up (censor). Clinical and medical history data came from the Duke Databank for Cardiovascular Disease, the data repository for the primary CATHGEN study.

All patients in CATHGEN had one 6-month follow-up and then annual follow-ups for all-cause mortality, with a maximum of 12 years of follow-up. Study staff adjudicated death events via National Death Index searches, supplementing with Social Security Death Index searches (Sutton et al., 2008).

Covariables for this project were measured for the CATHGEN clinical biorepository, as previously described (Wang et al., 2007). The following were dichotomous variables (yes/no) obtained by medical providers from the participants’ detailed medical history: smoking, type 2 diabetes, hyperlipidemia, and hypertension. Clinically defined continuous variables were BMI, creatinine, and ejection fraction.

### GWAS Genotyping

All GWAS genotyping was performed prior to this secondary data analysis project. Sample collection, processing, genotyping and quality control (QC) were performed for the primary CATHGEN study in the Molecular Genomic Core at the Duke Molecular Physiology Institute as previously described (Sutton et al., 2008; Kraus et al., 2015a,b). Post-quality-control genotype data were made available for this project via the Duke PEDIGENE^®^ biorepository database. Genotypes were called using Illumina’s GenomeStudio V2010.2 software (version 1.7.4 Genotyping module). SNPs with < 98% call frequency, MAF < 0.01 or that were out of Hardy–Weinberg equilibrium (*p* < 10^–6^) were excluded, resulting in 785,945 autosomal SNPs for our GWA screening analysis. Samples with < 98% call rates for all SNPs, gender mismatches, cryptic relatedness, or outlying ethnicity were excluded (172 samples). We performed secondary analyses of these GWAS data for our discovery and replication cohorts, testing variants having MAF > 0.01 among White CAD subjects.

### Statistical Analyses

Statistical analyses were performed with the R package using the following stepwise approach (R Core Development Team, 2013). We employed a two-step genome-wide screening, analyzing first the discovery and then the replication datasets for base- and clinical-covariate models. We then performed a combined meta-analysis of the two datasets.

We calculated means and frequencies for baseline demographic variables, diagnoses, and events. We defined time to event as the number of days from study enrollment (time at coronary catheterization and blood collection) to death from any cause. Data from surviving individuals were censored on the date of the last follow-up, consistent with our “survivorship with CAD” phenotype (Dungan et al., 2016). For our analyses of SNPs, we assumed an additive genetic model based on preliminary data demonstrating additive genetic effects (Dungan et al., 2016). We assigned wild-type genotype carriers a value of 0, heterozygous genotype carriers a value of 1, and homozygous carriers of the minor allele a value of 2 (Balding, 2006).

In order to determine CAD-specific genetic effects on survival, we employed Cox multivariate regression models to estimate instantaneous risk (hazard) of all-cause mortality among individuals with CAD by genotype group. For the initial screen, we fit a minimally adjusted (base) model controlling for age, sex, and four principal components of ancestry observed within this White sample. Additional models adjusted for clinical variables controlled for BMI, history of smoking, type 2 diabetes, hyperlipidemia, hypertension, creatinine, and ejection fraction. We used the same analyses in the replication cohort. *P*-values from suggestive discovery and replication results were meta-analyzed via the METAL combined z-score approach (Willer et al., 2010). Only variants that showed the same direction of effect in the discovery and replication phases were included in the meta-analysis phase. Our goal was to identify the strongest candidates with the most consistent findings across the three phases. For top variants, we used Kaplan-Meier curves to show survival probabilities by genotype.

#### Statistical Screening Thresholds

Our target association level was the standard GWAS threshold, *p* × 10^–8^. Where this stringent threshold was not met, we accepted variants meeting *p* × 10^–4^ or less in the discovery phase, indicating suggestive associations for candidate discovery. In the subsequent replication and meta-analysis phases, our goal was to set the association threshold based on Bonferroni correction for the number of variants tested within that phase. The target Bonferroni threshold for the replication phase was *p* < 5.3 × 10^–4^ (93 variants tested) and for the meta-analysis phase, *p* < 1.6 × 10^–3^ (all 30 tested variants).

## Results

### Demographics

We present demographic characteristics in Table 1. The discovery dataset had more male individuals than the replication dataset, but were similar in all other characteristics. Within the discovery and replication datasets, non-surviving individuals were, on average, older, had more severe CAD (greater CAD index), had a slightly higher prevalence of diabetes and hypertension, and had worse cardiac ejection fraction and renal function (creatinine levels) compared to their survived counterparts.

**TABLE 1:** Participant characteristics.

	Discovery dataset White CAD cases (684)		Replication dataset White CAD cases (404)	
	Alive (525)	Dead (159)	Alive (284)	Dead (120)
Age ± SD, (range)	63.32 ± 10.66	69.59 ± 10.78	56.99 ± 9.73	64.43 ± 11.1
CAD index ± SD	51.32 ± 17.72	58.29 ± 20.07	49.48 ± 17.79	54.88 ± 19.54
Male, n (freq)	388 (0.74)	122 (0.77)	152 (0.54)	59 (0.49)
BMI ± SD	29.52 ± 6.39	29.01 ± 7.12	30.62 ± 6.67	30.12 ± 6.11
Smoking, n (freq)	272 (0.52)	85 (0.53)	178 (0.63)	70 (0.58)
Dyslipidemia, n (freq)	356 (0.68)	108 (0.68)	216 (0.76)	87 (0.73)
T2DM, n (freq)	153 (0.29)	57 (0.36)	89 (0.31)	54 (0.45)
Hypertension, n (freq)	357 (0.68)	113 (0.71)	206 (0.73)	91 (0.76)
Ejection fraction ± SD	56.55 ± 12.39	50.37 ± 15.11	58.05 ± 10.77	51.51 ± 14.57
Creatinine ± SD	1.11 ± 0.66	1.51 ± 1.23	0.94 ± 0.21	1.17 ± 0.76
History of MI, n (freq)	175 (0.33)	77 (0.48)	111 (0.39)	64 (0.53)
History of ICC, n (freq)	145 (0.28)	55 (0.35)	72 (0.25)	39 (0.33)
History of CABG, n (freq)	185 (0.35)	78 (0.49)	78 (0.27)	49 (0.41)

*CAD, coronary artery disease; SD, standard deviation; BMI, body mass index; T2DM, Type 2 diabetes mellitus; MI, myocardial infarction (ever); ICC, intra-coronary procedures (percutaneous coronary transluminal angiography/stent); CABG, coronary artery bypass graft.*

### Follow-Up Events

In the discovery dataset (*n* = 684), the median follow-up time was 2,004 days (5.5 years) and maximum follow-up was 3,953 days (10.8 years). At the time of analysis, 159 individuals (23.3%) were deceased on follow-up. In the replication dataset (*n* = 404), the median follow-up was 3,326 days (9.1 years) and the maximum follow-up was 4,420 days (12 years). At the time of analysis, 120 individuals (29.7%) from the replication cohort were deceased on follow-up.

### Genome-Wide Variants Associated With Survival Among Individuals With CAD

**FIGURE 2:**
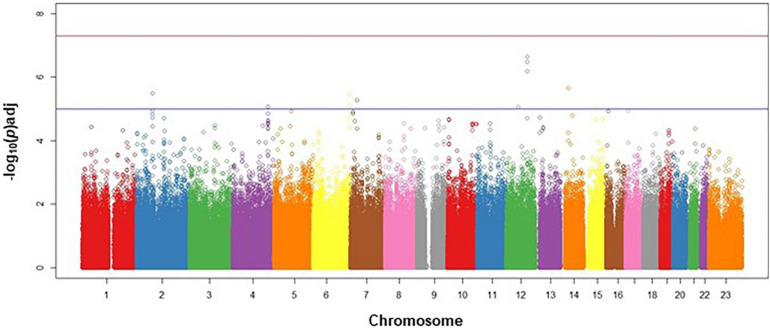
Manhattan plot for discovery phase. Manhattan plot of discovery phase SNPs in genomic order by chromosome and position on the chromosome (*X*-axis), plotted against the negative log *(adj)p*-value of each SNP’s association with survival events in CAD cases, adjusted for age, sex, and ancestry. Lower horizontal (blue line) represents *p* = 10^–5^; upper horizontal (red) line represents *p* = 5×10^–8^.

The discovery analysis Q-Q plot indicated that our observed genome-wide signal was consistent with the expected distribution under the null hypothesis (Supplementary Figure 1). The Manhattan plot (Figure 2) shows the negative log p-value for each SNP by chromosome. Given the exploratory aim of this genome-wide screen, we have chosen to report the top variants at each phase of the analysis that had consistent directions of effect across the discovery and replication phases.

#### Discovery

No SNPs met the standard 10^–8^ *p*-value threshold (Supplementary Table 1) for the base model analyzed within the discovery dataset; therefore, we moved forward 93 SNPs that had suggestive associations (*p* × 10^–4^ or less) with all-cause mortality (Supplementary Table 2). After controlling for clinical covariates, we found that the top three discovery variants (rs7138358, rs7305831, and rs12579455) achieved the accepted genome-wide threshold of *p* × 10^–8^ (Table 2). These three variants mapped to the same gene region on chromosome 12q21 (*MIR1251/RMST*) and were determined to be in linkage disequilibrium, having *r*^2^ > 0.85 in European Americans (Machiela and Chanock, 2018).

**TABLE 2:** Clinically adjusted hazards of all-cause mortality among CAD cases for discovery-screened SNPs.

SNP	Allelic Variation	Chr:loc	Variant	MAF	Gene	*p*-value^†^			HR	*95%* CI	*Z*-score
						Discovery	Replication	Meta-analysis			
**Variants with Negative Direction of Effect (HR < 1)**											
rs587936	T > **A**,***C***,**G**	9:121582553	Intronic	0.38	*DAB2IP*	**4.79 × 10^–4^**	**0.02**	**2.25 × 10^–5^**	0.65	0.51–0.83	-4.24
rs118207	C > **T**	2:33112067	Intronic	0.42	*LTBP1*	**4.43 × 10^–4^**	0.32	**7.11 × 10^–4^**	0.66	0.52–0.83	-3.39
rs9531515	T > **A**,**G**	13:31380652	Intergenic	0.43	*B3GALTL/RXFP2*	**9.43 × 10^–6^**	0.40	**5.78 × 10^–5^**	0.56	0.44–0.72	-4.02
rs31269	G > **A**,**C**	5:76330127	Intronic	0.50	*SV2C*	**2.35 × 10^–5^**	0.41	**1.17 × 10^–4^**	0.60	0.47–0.76	-3.85
rs349443	G > **T**	1:41811725	Intronic	0.38	*HIVEP3*	**7.19 × 10^–5^**	0.56	**4.68 × 10^–4^**	0.59	0.45–0.76	-3.50
rs242413	C > **T**	14:56161083	Intronic	0.31	*PELI2*	**7.67 × 10^–7^**	0.71	**3.35 × 10^–5^**	0.49	0.36–0.65	-4.15
rs7069959	**T** > C,G	10:130871742	Intergenic	0.27	*GLRX3/MIR378C*	**1.85 × 10^–4^**	0.76	**1.63 × 10^–3^**	0.57	0.43–0.77	-3.15
**Variants With Positive Direction Of Effect (HR > 1)**											
rs13007553	**T** > C	2:3058976	Intronic	0.36	*LINC01250*	**1.07 × 10^–3^**	**0.03**	**9.53 × 10^–5^**	1.47	1.17–1.86	3.90
rs13022539	**A** > G,T	2:3058283	Intronic	0.49	*LINC01250*	**9.15 × 10^–5^**	0.15	**7.08 × 10^–5^**	1.59	1.26–2.01	3.97
rs2108258	A > **G**	7:20752375	Intronic	0.08	*ABCB5*	**1.12 × 10^–4^**	0.16	**8.61 × 10^–5^**	1.89	1.37–2.61	-3.93
rs17164717	A > **G**	7:11527743	Intronic	0.02	*THSD7A*	**1.77 × 10^–6^**	0.12	**2.13 × 10^–6^**	4.33	2.37–7.90	-4.74
rs10240390	A > **C**,**G**	7:11527648	Intronic	0.02	*THSD7A*	**2.27 × 10^–6^**	0.21	**6.40 × 10^–6^**	4.28	2.34–7.83	-4.51
rs1865093	C > **T**	19:39445494	2Kb upstream	0.17	*SUPT5H*	**6.33 × 10^–4^**	0.26	**6.79 × 10^–4^**	1.66	1.24–2.23	3.40
rs7037490	**A** > C,G	9:38085787	Intergenic	0.16	*SHB/ALDH1B1*	**1.07 × 10^–3^**	0.28	**1.12 × 10^–3^**	1.59	1.20–2.10	3.26
rs7305964	**G** > A	12:97499951	Intronic	0.32	*RMST*	**1.00 × 10^–5^**	0.31	**3.78 × 10^–5^**	1.71	1.35–2.18	-4.12
rs17009399	G > **C**	2:73829186	5-prime UTR	0.08	*STAMBP*	**9.30 × 10^–5^**	0.35	**2.44 × 10^–4^**	2.01	1.42–2.85	-3.67
rs744680	G > **A**	10:129943431	Intronic	0.20	*EBF3*	**2.78 × 10^–5^**	0.38	**1.12 × 10^–4^**	1.74	1.34–2.25	3.86
rs12620516	G > **C**,**T**	2:216553597	Intergenic	0.29	*RPL37A/IGFBP2*	**7.40 × 10**^–^**^4^**	0.42	**1.58 × 10^–3^**	1.50	1.19–1.90	3.16
rs1865090	T > **A**,**C**	19:39441383	Intergenic	0.17	*RPS16/SUPT5H*	**7.40 × 10^–4^**	0.43	**1.62 × 10^–3^**	1.65	1.23–2.22	-3.15
rs4802033	G > **A**	19:39489326	Intergenic	0.17	*TIMM50*	**3.71 × 10^–4^**	0.46	**1.06 × 10^–3^**	1.69	1.27–2.26	3.27
rs7305831	T > **C**	12:97490908	Intronic	0.46	*RMST*	4.30 × 10^–8^*	0.48	**1.80 × 10^–6^**	1.98	1.55–2.52	4.78
rs7138358	**C** > T	12:97491385	Intronic	0.46	*RMST*	5.03 × 10^–8^*	0.51	**2.29 × 10^–6^**	1.97	1.55–2.52	-4.73
rs10819587	G > **A**	9:99019019	Intronic	0.11	*COL15A1*	**1.82 × 10^–4^**	0.58	**9.62 × 10^–4^**	1.80	1.32–2.45	3.30
rs4150403	C > **T**	2:127292492	Intronic	0.09	*ERCC3*	**5.62 × 10^–5^**	0.58	**4.13 × 10^–4^**	2.04	1.44–2.89	3.53
rs2297603	G > **A**,**T**	9:99015983	Exonic-missense	0.11	*COL15A1*	**2.49 × 10^–4^**	0.59	**1.22 × 10^–3^**	1.77	1.31–2.41	3.23
rs12579455	A > **G**,**T**	12:97481554	Intronic	0.46	*RMST*	7.927 × 10^–8^*	0.60	**4.82 × 10^–6^**	1.95	1.53–2.49	4.57
rs12515837	T > **A,C**	5:30940209	Intergenic	0.05	*LOC729862/CDH6*	**2.75 × 10^–4^**	0.64	**1.51 × 10^–3^**	2.21	1.44–3.39	-3.17
rs17009433	T > **C**	2:73872884	Intronic	0.10	*STAMBP*	**9.15 × 10^–5^**	0.70	**4.13 × 10^–3^**	1.87	1.37–2.55	-2.87
rs896651	**T** > A,G	18: 61564558	Intergenic	0.35	*CDH20/RNF152*	**2.46 × 10^–5^**	0.70	**3.46 × 10^–4^**	1.66	1.31–2.10	3.58
rs11126419	C > **T**	2:73864992	Intronic	0.09	*STAMBP*	**4.29 × 10^–5^**	0.72	**5.32 × 10^–4^**	2.07	1.46–2.94	3.46

Less frequent (minor) allele bolded. Bold numerals indicate *p* < 0.05. *indicates GWAS significance threshold *p* < 10^–8^. ^†^Results for statistical model controlling for age, sex, and four principal components of ancestry (base model) plus additional clinical covariates: body mass index (BMI), history of smoking, type 2 diabetes, hyperlipidemia, hypertension, creatinine, and ejection fraction. SNP, single nucleotide polymorphism; Chr, chromosome; loc, genomic location (GRCh38.p12); MAF, minor allele frequency; HR, hazard ratio; CI, confidence interval; UTR, untranslated region; Kb, kilobase.

#### Replication

Neither base models nor clinically adjusted models produced SNPs meeting our target Bonferroni threshold for replication of *p* < 5.3 × 10^–4^. The top two discovery SNPs that had consistent directions of effect in both cohorts met nominal significance (*p* < 0.05) for replication (rs13007553 and rs587936; Table 2). The *RMST* discovery variants that met the threshold of adjusted *p* × 10^–8^ (rs7138358, rs7305831, and rs12579455) did not achieve replication threshold of *p* < 5.3 × 10^–4^; however, they did meet the meta-analysis threshold of *p* < 1.6 × 10^–3^.

We observed only nominal significance levels (*p* < 0.05) in the replication, causing concern for the suitability of the chosen replication dataset. In order to evaluate for the congruence of SNP effects between both datasets, we generated an effect size scatterplot (Supplementary Figure 2) for the SNPs having a MAF > 0.05, adjusted *p* < 0.05 and HR < 40 (1,229 SNPs), as these represented the most stable exploratory results. Specifically, we plotted each SNP’s HR for the discovery dataset on the x-axis and their HR for the replication dataset on the y-axis. Supplementary Figure 2 shows multiple SNPs having *p* < 0.05 in both datasets with congruent effect sizes between both the discovery and replication datasets (red circles). Supplementary Table 3 contains the detailed results for the 1,229 SNPs from Supplementary Figure 2.

#### Meta-Analysis

Of the discovery-identified SNPs, 30, representing 21 distinct gene regions, met the threshold for significance in our meta-analysis *p* < 1.6 × 10^–3^ and had consistent directions of effect across the two cohorts (Table 2). Only two of these variants (mapped to the same gene) were rare (MAF = 0.02); all other variants were common in the combined sample with MAF > 0.05. Only two variants were significant across all analyses (discovery, replication, and meta-analyses) — one demonstrating decreased and the other increased risk for all-cause mortality, adjusted for clinical covariates (Table 2).

**FIGURE 3:**
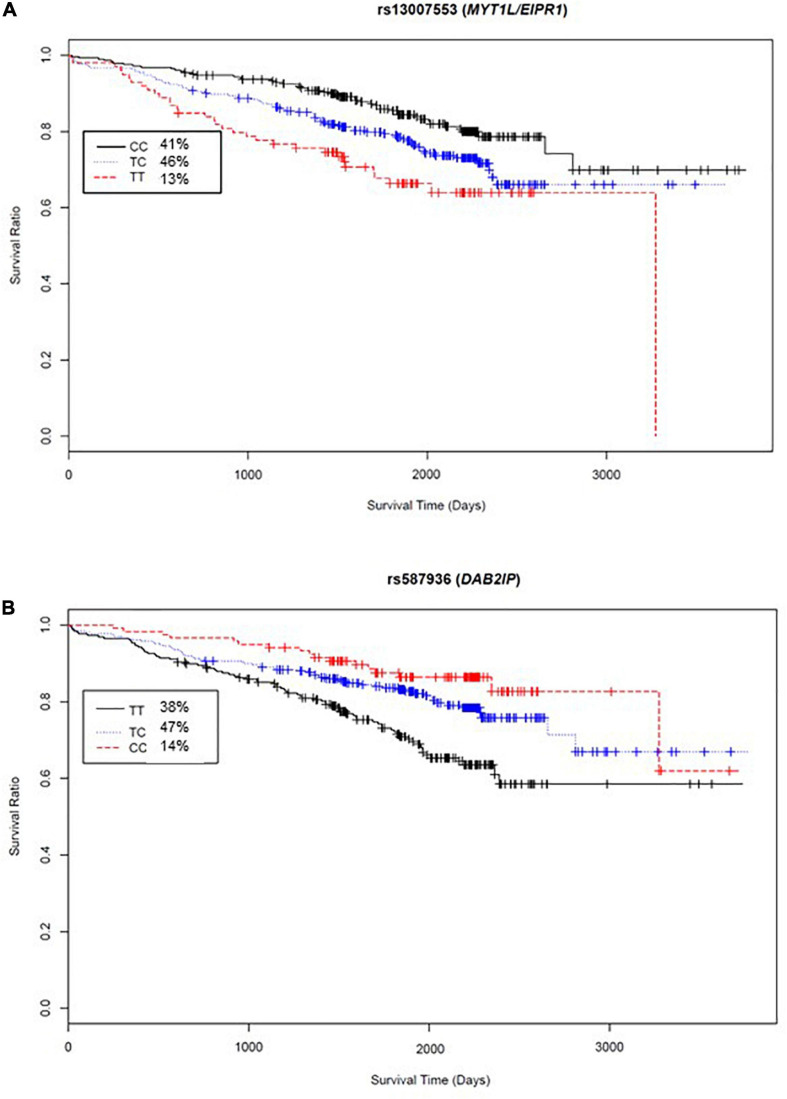
Kaplan-Meier curves for top two meta-analyses genome-wide SNP exemplars. *X*-axis, days from enrollment to death or last follow up; *Y*-axis, probability of survival; inset box, genotype frequencies. **(A)** Survival curve by rs13007553 genotype indicates 1.47 times increased hazard of all-cause mortality for each addition of the minor allele (T nucleotide). **(B)** Survival curve by rs587936 genotype indicates reduced hazard of all-cause mortality with each addition of the minor allele (C nucleotide) (HR 0.65).

The variant significantly associated with increased risk by genotype was rs13007553. Among White CAD cases, each copy of the minor *T* allele for rs13007553 conferred 53% increased risk of all-cause mortality (Figure 3A) in the clinically adjusted model (HR 1.47, 95% CI 1.17–1.86, *p(adj)* = 1.07 × 10^–3^ [discovery], *p(adj)* = 0.03 [replication], *p(adj)* = 9.53 × 10^–5^ [meta-analysis]). The rs13007553 SNP is located in *LINC01250*, or long intergenic non-protein coding RNA 1250. This common intronic SNP (MAF = 0.36) on Chromosome 2p25.3 (GRCh38.p12 build position 3058976) resides between *MYT1L* and *EIPR1* (alias *TSSC1*). Another intergenic variant for *LINC01250* (*MYT1L/EIPR1;* rs13022539) was significant in the discovery dataset (*p* = 6.03 × 10^–5^; *p(adj)* = 9.15 × 10^–5^) and meta-analysis (*p* = 7.08 × 10^–5^) but did not reach significance in the replication dataset (*p* = 0.15). However, these two SNPs are in linkage disequilibrium among European samples (*r*^2^ = 0.53, *D*’ = 0.96, *p* < 0.0001) indicating that they may represent the same underlying genetic effect (Machiela and Chanock, 2018). There is no functional annotation for rs13007553, and it has no expression quantitative trail loci (eQTL) relationships according to the Genotype-Tissue Expression project (GTEx).

Conversely, rs587936 demonstrated attenuated risk by number of minor alleles. In our analysis, each copy of the minor C allele for rs587936 conferred significantly reduced risk of all-cause mortality (Figure 3B; HR 0.65, 95% CI*0.51*-0.83, *p(adj)* = 4.79 × 10^–4^ [discovery], *p(adj)* = 0.02 [replication], *p(adj)* = 2.25 × 10^–5^ [meta-analysis]), controlling for clinical covariates. The rs587936 marker is a common intronic SNP (MAF = 0.38) on Chromosome 9q33.2 (GRCh38.p12 build position 121582553) mapped to the first intron of *DAB2IP* (alias *AIP1*). There is no functional annotation for rs587936 and no evidence of expression quantitative trait loci (eQTL) with *DAB2IP* (GTEx database). However, further review of gene expression data in the GTEx database shows modest evidence that rs587936 is associated with expression of the pseudogene *GGTAP1* in heart tissue and expression of an uncharacterized locus, *LOC102723324*, in multiple tissues including adipose, lung, and aorta.

## Discussion

In the present study, we conducted genome-wide discovery, replication, and meta-analysis screening for genetic contribution to differential survival outcomes among 1,088 White patients with clinically significant CAD from the southeastern United States. We observed improved *p*-values after controlling for the clinical covariates but only minimal shifts in the effect sizes. For this reason, we discuss the clinically adjusted models here and provide the base-adjusted results as Supplementary Material. Our major findings are the identification of two common gene variants consistently associated with risk for all-cause mortality among White patients with CAD. The minor C allele for *DAB2IP (AIP1)* rs587936 was associated with significantly reduced risk of all-cause mortality; whereas, the minor T allele for rs13007553, residing between the *MYTIL* and *EIPR1* genes, conferred significantly increased risk for all-cause mortality.

While our findings offer substantive evidence of an association between rs587936 (HR < 1) and survival outcomes among White individuals with CAD, van der Harst and Verweij (2018) reported that *DAB2IP* rs885150 was associated with reduced risk of CAD (OR < 1) in a recent GWAS of UK Biobank and CARDIoGRAMplusC4D (OR0.96, 95% CI0.95–0.98, *p* = 2.1 × 10^–8^). In prior studies, researchers had reported *DAB2IP* association with lower-limb ischemia and CAD case status (Harrison et al., 2012; Smith and Newton-Cheh, 2015), and with aortic aneurysm (Gretarsdottir et al., 2010), all of which increase mortality risk in patients with CAD. The most commonly studied variant of *DAB2IP*, rs7025486, is associated with cardiac risk phenotypes and is part of a 21-gene risk score that significantly predicts CAD case status. It was significantly associated with blood lipid levels in a cohort of Pakistani people, but was not an independent predictor as a single SNP (Shahid et al., 2017). We determined that rs7025486 and rs587936 are not in linkage disequilibrium (*r*^2^ = *0.0001, d’* = *0.0145*, *p* = *0.547*), suggesting allelic heterogeneity in this region (Machiela and Chanock, 2018).

*DAB2IP* is a ras/GAP tumor suppressor gene that encodes an ASK1-interacting protein abundantly expressed in vascular endothelial cells (Zhang et al., 2015). *DAB2IP* is an alias for *ASK-1*, which is broadly termed *AIP1* (anti-inflammatory protein-1) for its so-named properties (Zhang et al., 2015). *In vitro* and murine knockout studies support AIP1 as “a signaling adaptor molecule implicated in stress and apoptotic signaling induced by proinflammatory mediators” (Huang et al., 2013, p. 795). *DAB2IP*-encoded ASK1/AIP1 also suppresses atherosclerosis by limiting hyperlipidemia-induced inflammation and vascular endothelial dysfunction (Huang et al., 2013). These reported relationships between *DAB2IP* and vascular anti-inflammatory processes offer biologic plausibility for gene variation in *DAB2IP* conferring increased survival time for people with CAD. Additional replication and validation are required to test hypotheses about the functional relation of *DAB2IP* on survival.

Consistent with its earlier recognition as a ras/GAP tumor suppressor, *DAB2IP* also is associated with risk for various cancers and cancer cell proliferation and migration (Zhou et al., 2015; Li et al., 2016; Olsen et al., 2017). Among its effects, *DAB2IP* inhibits oncogenic processes and facilitates cancer cell apoptosis (Liu et al., 2016). In our earlier study in which we used a candidate gene approach, we found the tumor suppressor gene *LSAMP* to be a statistically significant marker of survivorship with CAD (Dungan et al., 2013). Our present identification of an association between another tumor-suppressor gene, *DAB2IP*, and CAD survival outcomes in the present GWA screening underscores the importance of considering antagonistic pleiotropy when seeking to identify genetic effects in complex diseases. Antagonistic pleiotropy refers to a gene having dual effects such that it may confer both enhanced fitness via effects on a beneficial trait and increased risk for a detrimental trait. Murine knockout studies provided early evidence that *DAB2IP* genetic effects may be pleiotropic. Both global gene knockouts and deletion of *DAB2IP* specifically in vascular endothelial cells consistently resulted in enhanced inflammatory responses, atherosclerosis exacerbation, and graft arteriosclerosis progression (Zhang et al., 2015). However, *DAB2IP* loss also conferred resistance to prostate cancer through modulation of apoptosis (Zhou et al., 2015). Theoretically, a mouse having no ability to produce *DAB2IP* might simultaneously develop hallmark signs of CAD (inflammation, athero- and arteriosclerosis) and protection against the development of prostate cancer. It is unclear whether antagonistic pleiotropy in animal models extrapolates to humans. However, other examples of antagonistic genetic effects involving CAD and cancer risk exist in human cohort studies (Kulminski et al., 2011). Further work is needed to expand our understanding of the complex relationships among *DAB21P (AIP1)*, CAD, cancer, and survival.

The other variant consistently associated with survival in CAD in the present study, the intergenic rs13007553 (*LINC01250*) located between *MYT1L* and *EIPR1* (alias *TSSC1*), was linked with increased risk of all-cause mortality among CAD cases. The biologic relevance is unclear regarding these genes nearby to *MYT1L* and our CAD survival phenotype. Similar to the candidate gene *LSAMP* identified in our prior studies of survival with CAD (Dungan et al., 2013, 2016), *MYT1L* plays a key role in neuronal differentiation. Variation in *MYT1L* is implicated in fibromyalgia and is associated with a non-specific clinical phenotype that includes intellectual disability, early-onset obesity, and speech delay (Docampo et al., 2014; Al Tuwaijri and Alfadhel, 2019). *EIPR1* (alias *TSSC1*, the other gene flanking rs13007553), is involved in endosomal retrieval pathways (Gershlick et al., 2016). *In vitro* experiments have demonstrated that *TSSC1* can regulate metastatic properties in breast cells (Wang et al., 2013). Our findings in the present study add evidence to the literature regarding the association of rs13007553 (i.e., the *MYT1L/EIPR1* intergenic region) with CAD-related survival outcomes.

Additional genes of interest in our results are rhabdomyosarcoma 2 associated transcript (*RMST)* and thrombospondin type-1 domain-containing protein 7A (*THSD7A*). Specifically, four *RMST* SNPs (rs7305964, rs7305831, rs7138358, and rs12579455) were associated with increased risk of all-cause mortality in the White CAD patients we analyzed (Table 2). The latter three SNPs were our top discovery hits. Models adjusted for clinical covariates for these three variants resulted in *p*-values meeting the stringent 10^–8^ threshold for significance. In a previous study, researchers reported a significant association between another GWAS-identified *RMST* SNP, rs10777845, and increased risk of sudden cardiac arrest among patients with CAD (OR 1.12, 95% CI 1.07–1.17; *p* = 5.0 × 10^–6^; Li et al., 2018). The mechanisms for *RMST* contribution to cardiovascular diseases are unknown. Another two of the SNPs with a significant association to CAD survival in the discovery phase of the present study annotate to *THSD7A* (rs17164717, rs10240390). *THSD7A* variation was associated with CAD in a GWAS of over 21,000 Han Chinese participants (rs17165136; OR 1.28, 95% CI 1.21–1.35; *p* < 1.00 × 10^–25^; Aouizerat et al., 2011). Subsequent functional studies with knockdown of *THSD7A* have demonstrated reduced monocyte adhesion via decreased expression of intracellular adhesion molecule-1 (ICAM-1), L-selectin, and integrin subunit beta 2 (ITGB2) (Li et al., 2018). The present study offers supportive evidence for *THSD7A* and *RMST* candidacy in cardiovascular disease.

Given the potential for shared genetic variation among phenotypes for survivorship with CAD and longevity, we also assessed whether our top hits with increased survival time (HR < 1) had previous associations with longevity phenotypes. We found no such associations in the NHGRI-EBI Catalog of human GWASs. Nor were the loci of these variants near the most-validated longevity candidate regions (Pilling et al., 2017; Deelen et al., 2019; Zenin et al., 2019).

## Strengths and Limitations

Results of the present exploratory genome-wide screen add new insights to the limited literature on the associations of CAD candidate genes with survival outcomes, most notably regarding improved survival outcomes. We found only one other phenotypically similar candidate study in the literature. In that study, researchers reported that *SDF1* (alias, *CXCL12*) conferred significantly improved event-free survival among patients with symptomatic CAD, even after controlling for clinical covariates (Rath et al., 2016).

Our results also add to the literature on the genetic candidates for increased risk for mortality in CAD. The most rigorous candidate evidence to date comes from a resequencing of *APOA1* in which investigators identified a novel mutation that predicted a 2.5-fold increased hazard of death with a mean reduction of 10 years in survival time in heterozygous carriers among participants in the Copenhagen City Heart Study, who were followed for 31 years (Haase et al., 2011).

Our use of an extensive clinical cardiovascular biorepository having genome-wide participant data and a maximum of 12 years of longitudinal follow-up was both a strength and a limitation of the present study. While this resource allowed us to explore genomic variation associated with longitudinal survival outcomes among people with prevalent CAD, the medical history variables were not defined and measured from a research perspective, limiting our ability to explore the effects of these factors. For example, smoking history was operationalized as a self-reported “yes/no” variable obtained by provider intake assessment rather than being measured for optimal internal validity using additional variables such as ever-/current, frequency, and exposure type or interval (Florescu et al., 2009). Additionally, dietary variables were not measured in CATHGEN. Given the importance of smoking and diet as risk factors for CAD (McGillicuddy and Roche, 2012; Gambardella et al., 2017) and as having potential gene-interaction effects (Horne et al., 2009), these gaps in the database are a limitation for the present study.

Because our goal in the present study was to add to the literature with an exploratory GWA screen for the CAD survival phenotype, we decided to relax the *p*-value thresholds and focus on the top-most variants with consistent directions of effect across the analysis phases. We readily acknowledge that our decision to relax the *p*-value thresholds for the discovery and replication phases represents a limitation. We offer two points of consideration: First, our concern about this limitation prompted us to evaluate the 1,229 most stable SNPs (MAF > 0.05, adjusted *p*-values < 0.05 and HR < 40) for the presence and quality of effect size congruence between both of the datasets (Supplementary Figure 2). The scatterplot demonstrates the extent to which the SNP effects correlate between the discovery and replication analyses. We are encouraged by the numerous SNPs having similar effect sizes in the replication dataset as they had in the discovery dataset (red circles). Second, Purcell et al. (2009) set an important precedent for relaxing discovery *p*-values when SNPs for schizophrenia identified in the discovery phase of their GWAS did not meet the stringent 10^–8^ GWAS *p*-value threshold, yet were later replicated and also demonstrated significance as part of a genomic risk score for schizophrenia and other related phenotypes (Marees et al., 2018). Future studies designed specifically for the aim of identifying genome-wide association with survival outcomes among patients with CAD should strive for the more stringent 10^–8^ *p*-value criterion typically expected for GWAS-level significance. Our results in the present study identify candidate genes for further investigation. To support such work, we offer details of variant annotations, effect sizes, and *p*-values in Supplementary Table 3. We continue to seek opportunities for additional meta-analyses and have constructed our results tables to facilitate such analyses.

Differences in clinical characteristics and study design between the discovery and replication cohorts are likely culprits in the limited number of replicated variants observed. The potential exists for confounding between SNP variation and untested clinical covariates and/or survival outcomes. Of note, CATHGEN CAD cases are defined by the Duke CAD index, a measure of CAD severity which accounts for both the extent and location of coronary stenosis. Since disease severity was used to determine case status in the original sub-studies, it would not have been prudent to use disease severity as a covariant in the models in the present study. Therefore, we could not evaluate the specific influence of disease severity on survival outcomes in the present sample. Similarly, we could not evaluate the specific influence of left main arterial stenosis on survival within the present study due to the limited number of individuals with this severe phenotype. Addressing this limitation in future research would improve upon our understanding of disease severity and stenosis location as mediators or moderators of genetic effects on survival outcomes. Additionally, our findings may represent genetic signal for unstudied factors that may co-occur among non-surviving patients with CAD.

Treatment effects may have confounded the survival rates in the CATHGEN clinical observational studies from which we drew the data for the present analysis. We were unable to control for medical history of myocardial infarction and interventional treatment via CABG or stent placement due to excessive missing data; attempting to include these variables in our statistical models resulted in model divergence. We were also unable to control for the use of medications such as aspirin and other antiplatelet agents, statins, and beta-blockers, which are well-established independent predictors of survival and mortality among CAD patients (Antithrombotic Trialists’ Collaboration, 2002; Dézsi and Szentes, 2017; Rodriguez et al., 2019). The observed association of rs587936 with improved survival in CAD is likely the most susceptible to confounding by medication effects of the findings in the present study. However, our analyses identified an association between rs13007553 and increased risk of mortality in CAD even with the presumed presence of medication treatment effects. We would expect that controlling for medication use would strengthen the genetic signal of that variant further.

The data source for the present secondary analysis did not include sufficient data to accurately adjudicate cardiac causes of death. As we discussed in a previous study, the most internally valid phenotype definition for survivorship with CAD is the time to death due to CAD-specific causes. In the absence of sufficient data on cause of death, we selected all-cause mortality as a suitable primary endpoint (Dungan et al., 2013). We also must consider the potential for survival bias in genetic-associated endpoints, as we have examined previously (Dungan et al., 2013). Simulated evidence of lethal cardiac events suggests that we can expect an erosion of no more than 20% in effect size due to survival bias since our cohort’s mean age is less than 75 years (Anderson et al., 2011). A more recent study concluded that survival bias was unlikely when genetic effects were small (OR, HR < 2) when examining recurrent events after non-fatal MI (Hu et al., 2017). In the present study, however, we did not study a conditional, recurrent endpoint.

Our results are only generalizable to White people with CAD in the southeastern United States. We readily acknowledge the need for expanding this work to include ancestrally diverse and marginalized individuals, as they are often at greater risk for both CAD and mortality. Expanding diverse representation for genomic studies is a priority for our future work, and we continue to explore solutions to address sampling and inclusion limitations.

## Conclusion

Our goal with the present study was to generate discovery variants as a logical next step to support future hypothesis-driven work. Using genome-wide screening, we identified two candidate gene markers associated with survival outcomes across a 12-year follow-up among 1,088 White participants whose CAD was clinically defined via cardiac catheterization. Allelic variation in rs587936 (*DAB2IP*) conferred reduced risk and rs13007553 conferred increased risk for all-cause mortality, even after controlling for clinical covariates. These findings extend prior findings of associations of *DAB2IP* with CAD phenotypes to include survival in those with CAD. Our observed association between rs13007553 (*LINC01250*, intergenic of *MYT1L/TSSC1*) and increased risk of all-cause mortality in patients with CAD is a novel finding among the current literature. These candidate variants do not appear to overlap with the top longevity candidate genes. Additional research is needed to identify genetic contributions to survivorship in those with significant CAD as well as the underlying biological mechanisms of those contributions. Our results will serve as a resource for *in silico* and meta-analyses and can inform the design of future studies. Such work could lead to a better understanding of mortality risk and protective mechanisms in the context of the coronary disease state.

## Data Availability Statement

The original contributions presented in the study are publicly available. These data can be found here: https://www.ncbi.nlm.nih.gov/projects/gap/cgi-bin/study.cgi?study_id= phs000703.v1.p1 [Accession number phs000703.v1.p1].

## Ethics Statement

The studies involving human participants were reviewed and approved by Duke University Medical Center Institutional Review Board. The patients/participants provided their written informed consent to participate in this study.

## Author Contributions

JD, XQ, EH, and WK contributed to conception and design of the study. CH (senior analyst) procured the database. MH adjudicated and managed data in preparation for analyses. XQ performed the statistical analysis. JD wrote the first draft of the manuscript. EH and WK contributed substantive edits for the manuscript. All authors contributed to manuscript revision, read, and approved the submitted version.

## Conflict of Interest

The authors declare that the research was conducted in the absence of any commercial or financial relationships that could be construed as a potential conflict of interest.

## Supplementary Material

Supplementary Figure 1 Discovery phase Q-Q plot.

Supplementary Figure 2 SNP effect size congruence between discovery and replication datasets.

Supplementary Table 1 Base model hazard of all-cause mortality among cad cases for discovery-screened SNPs. Less frequent (minor) allele bolded. Bold numerals indicate *p* < 0.05. ^∗^Results for base statistical model controlling for age, sex, and four principal components of ancestry. SNP, single nucleotide polymorphism; Chr, chromosome; loc, genomic location (Grch38.p12); MAF, minor allele frequency; HR, hazard ratio; CI, confidence interval; UTR, untranslated region; Kb, kilobase.

Supplementary Table 2 Top 93 discovery SNPs. ^∗^Base model adjusting for age, sex, and four principal components of ancestry; *p(adj)*, clinically adjusted model controlling for body mass index (BMI), history of smoking, type 2 diabetes, hyperlipidemia, hypertension, creatinine, and ejection fraction. Bold indicates GWAS-level significance *p* = 10^–8^.

Supplementary Table 3 Details of 1229 SNPs evaluated for effect size congruence.

## Abbreviations

*AIP1*: apoptosis signal regulating kinase 1
*ASK1*: apoptosis signal-regulating kinase 1
BMI: body mass index
CABG: coronary artery bypass graft
CAD: coronary artery disease
CATHGEN: catheterization genetics biorepository
CHD: coronary heart disease
CI: confidence interval
*DAB2IP*: disabled homolog 2 interacting protein
DNA: deoxyribonucleic acid
*EARP*: endosome-associated recycling protein
*EIPR1*: EARP complex and GARP complex interacting protein 1
GWAS: genome-wide association study
HR: hazard ratio
*LSAMP*: limbic system-associated membrane protein
MAF: minor allele frequency
MI: myocardial infarction
*MYT1L*: myelin transcription factor 1like
Ras/GAP: ras/GTPase activating protein
SNP: single nucleotide polymorphism
TSSC1: tumor-suppressing subtransferable fragment candidate gene 1.
