# Corrigendum: Genome-Wide Variants Associated With Longitudinal Survival Outcomes Among Individuals With Coronary Artery Disease

**DOI:** 10.3389/fgene.2021.726466

**Published:** 2021-07-27

**Authors:** Jennifer R. Dungan, Xue Qin, Melissa Hurdle, Carol S. Haynes, Elizabeth R. Hauser, William E. Kraus

**Affiliations:** ^1^Division of Healthcare in Adult Populations, School of Nursing, Duke University, Durham, NC, United States; ^2^School of Medicine, Duke Molecular Physiology Institute, Duke University, Durham, NC, United States; ^3^Department of Biostatistics and Bioinformatics, Duke University School of Medicine, Durham, NC, United States; ^4^Cooperative Studies Program Epidemiology Center, Durham VA Medical Center, Durham, NC, United States; ^5^Division of Cardiology, Department of Medicine, School of Medicine, Duke University, Durham, NC, United States

**Keywords:** coronary artery disease, survival analysis, genome-wide association study, age-related disease, candidate gene analyses

In the original article, there was a mistake in the total number of White CAD cases labeled for the Discovery group; this subsample was 684, not 1,099 as published. This mistake was represented throughout the article, including in Figure 1 (and Legend), Table 1, and the Abstract, Materials and Methods, Discussion, and Conclusion sections. As such, the following sample size corrections were made.

**Figure 1:**
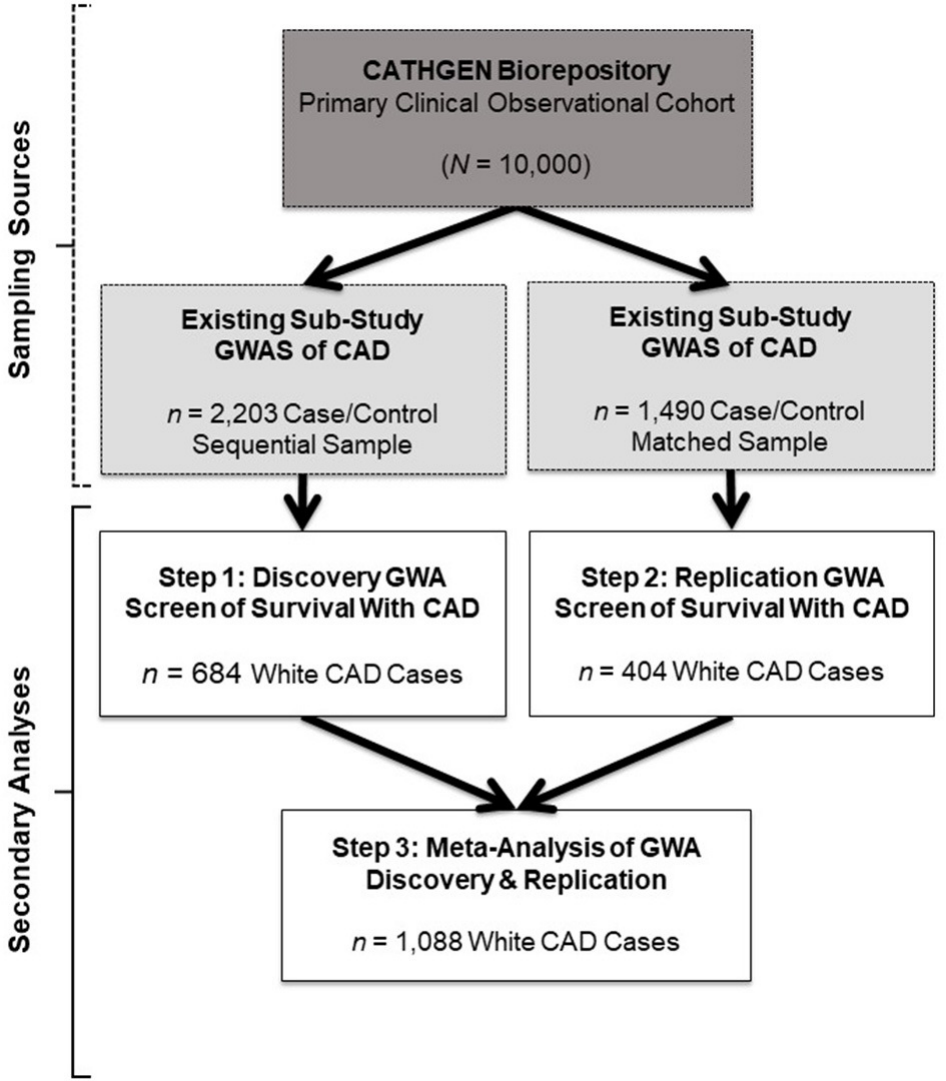
Study design and sample schema. This figure depicts the primary study (dark gray box) and extant sub-study data (light gray boxes) from which our retrospective datasets for the present analyses were derived (white boxes). The CATHGEN Biorepository containing data from 10,000 individuals recruited after cardiac catheterization (dark gray box) supplied the samples for two separate GWASs of CAD sub-studies (light gray boxes), providing the extant GWAS genotype data for our secondary analysis. The first GWAS sub-study contained data from a sequential sample of 2,203 CATHGEN CAD cases and controls (light gray box, left); the other CAD GWAS sub-study had data from 1,490 CATHGEN CAD cases and matched controls (light gray box, right). The white boxes display the secondary datasets we analyzed in the present retrospective study. Specifically, we derived our discovery cohort of 684 White CAD cases (white box, left) from the sequential case-control GWAS data; this discovery dataset was analyzed for Step 1. Our replication cohort of 404 White CAD cases (white box, right) was derived from the matched case-control GWAS dataset; this replication dataset was analyzed for Step 2. We then performed meta-analyses of our discovery and replication cohorts (black arrows converging on the bottom white box) for Step 3.

**Table 1:** Participant characteristics.

	**Discovery dataset White CAD cases (684)**		**Replication dataset White CAD cases (404)**	
	**Alive (525)**	**Dead (159)**	**Alive (284)**	**Dead (120)**
Age ± SD, (range)	63.32 ± 10.66	69.59 ± 10.78	56.99 ± 9.73	64.43 ± 11.1
CAD index ± SD	51.32 ± 17.72	58.29 ± 20.07	49.48 ± 17.79	54.88 ± 19.54
Male, n (freq)	388 (0.74)	122 (0.77)	152 (0.54)	59 (0.49)
BMI ± SD	29.52 ± 6.39	29.01 ± 7.12	30.62 ± 6.67	30.12 ± 6.11
Smoking, n (freq)	272 (0.52)	85 (0.53)	178 (0.63)	70 (0.58)
Dyslipidemia, n (freq)	356 (0.68)	108 (0.68)	216 (0.76)	87 (0.73)
T2DM, n (freq)	153 (0.29)	57 (0.36)	89 (0.31)	54 (0.45)
Hypertension, n (freq)	357 (0.68)	113 (0.71)	206 (0.73)	91 (0.76)
Ejection fraction ± SD	56.55 ± 12.39	50.37 ± 15.11	58.05 ± 10.77	51.51 ± 14.57
Creatinine ± SD	1.11 ± 0.66	1.51 ± 1.23	0.94 ± 0.21	1.17 ± 0.76
History of MI, n (freq)	175 (0.33)	77 (0.48)	111 (0.39)	64 (0.53)
History of ICC, n (freq)	145 (0.28)	55 (0.35)	72 (0.25)	39 (0.33)
History of CABG, n (freq)	185 (0.35)	78 (0.49)	78 (0.27)	49 (0.41)

*CAD, coronary artery disease; SD, standard deviation; BMI, body mass index; T2DM, Type 2 diabetes mellitus; MI, myocardial infarction (ever); ICC, intra-coronary procedures (percutaneous coronary transluminal angiography/stent); CABG, coronary artery bypass graft*.

In Figure 1, the total number of White CAD cases labeled in the Discovery Step 1 box is 684, not 1,099. This changes the total number of White CAD cases listed in the Meta-Analysis Step 3 box to 1,088, not 1,503. Also, in the Figure 1 Legend, the total number of White CAD cases is 684, not 1,099. The corrected Figure 1 and the legend appears below.

In Table 1, the sample size for the Discovery dataset is mislabeled for White CAD cases (*n* = 684, not 1,099 as published). Subsequently, the Alive/Dead sample numbers for the Discovery White CAD cases are also mislabeled: (Alive *n* = 525, not 883; Dead *n* = 159, not 216 as published). This was a mislabeling mistake; all other reported values in the table have been verified. The corrected Table 1 appears below.

A correction has been made to the Abstract, Approach and Results:

“We performed discovery (*n* = 684), replication (*n* = 404), and meta-analyses (*n* = 1,088) for association of genomic variants with survival outcome using secondary data from White participants with CAD from two GWAS sub-studies of the Duke Catheterization Genetics Biorepository. We modeled time from catheterization to death or last follow-up (median 7.1 years, max 12 years) using Cox multivariable regression analysis. Target statistical screening thresholds were *p* × 10^−8^ for the discovery phase and Bonferroni-calculated *p*-values for the replication (*p* < 5.3 × 10^−4^) and meta-analysis (*p* < 1.6 × 10^−3^) phases. Genomewide analysis of 785,945 autosomal SNPs revealed two SNPs (rs13007553 and rs587936) that had the same direction of effect across all three phases of the analysis, with suggestive *p*-value association in discovery and replication and significant meta-analysis association in models adjusted for clinical covariates. The rs13007553 SNP variant, *LINC01250*, which resides between *MYTIL* and *EIPR1*, conferred increased risk for all-cause mortality even after controlling for clinical covariates [HR 1.47, 95% CI 1.17–1.86, *p(adj)* = 1.07 × 10^−3^ (discovery), *p(adj)* = 0.03 (replication), *p(adj)* = 9.53 × 10^−5^ (meta-analysis)]. *MYT1L* is involved in neuronal differentiation. *TSSC1* is involved in endosomal recycling and is implicated in breast cancer. The rs587936 variant annotated to *DAB2IP* was associated with increased survival time [HR 0.65, 95% CI 0.51–0.83, *p(adj)* = 4.79 × 10^−4^ (discovery), *p(adj)* = 0.02 (replication), *p(adj)* = 2.25 × 10^−5^ (meta-analysis)]. *DAB2IP* is a ras/GAP tumor suppressor gene which is highly expressed in vascular tissue. *DAB2IP* has multiple lines of evidence for protection against atherosclerosis.”

A correction has been made to the Materials and Methods section, sub-section Design:

“We conducted a secondary analysis of existing data from two separate GWAS sub-studies of participants sampled from the Catheterization Genetics study clinical cardiovascular biorepository (CATHGEN; *N* = 9,334; Sutton et al., 2008; Kraus et al., 2015a,b). We employed a two-step genome-wide association screen for variants associated with survival outcomes in patients with CAD, using the separate GWAS sub-studies for discovery (*N* = 684) and replication (*N* = 404).”

Another correction has been made to the Materials and Methods section, subsection Inclusion Criteria, Paragraph 2:

“We selected CAD-defined cases from the larger of the two sub-studies to serve as our GWAS discovery dataset (*n* = 684). As in the primary CATHGEN study, we defined positive CAD case status as having a Duke CAD index ≥ 32 (at least one vessel having at least 75% stenosis) determined by clinical coronary heart catheterization (Sutton et al., 2008). Of note, the Duke CAD index reflects both the extent and location of stenosis. It is used as an indicator of disease severity and includes individuals with left main coronary disease. Likewise, we selected CAD cases from the smaller GWAS sub-study to serve as the replication dataset (*n* = 404).”

A correction has also been made to the Discussion, Paragraph 1:

“In the present study, we conducted genome-wide discovery, replication, and meta-analysis screening for genetic contribution to differential survival outcomes among 1,088 White patients with clinically significant CAD from the southeastern United States. We observed improved *p*-values after controlling for the clinical covariates but only minimal shifts in the effect sizes. For this reason, we discuss the clinically adjusted models here and provide the base-adjusted results as Supplementary Material. Our major findings are the identification of two common gene variants consistently associated with risk for all-cause mortality among White patients with CAD. The minor C allele for *DAB2IP (AIP1)* rs587936 was associated with significantly reduced risk of all-cause mortality; whereas, the minor T allele for rs13007553, residing between the *MYTIL* and *EIPR1* genes, conferred significantly increased risk for all-cause mortality.”

Furthermore, a correction has been made to the Conclusion:

“Our goal with the present study was to generate discovery variants as a logical next step to support future hypothesis-driven work. Using genome-wide screening, we identified two candidate gene markers associated with survival outcomes across a 12-year follow-up among 1,088 White participants whose CAD was clinically defined via cardiac catheterization. Allelic variation in rs587936 (*DAB2IP*) conferred reduced risk and rs13007553 conferred increased risk for all-cause mortality, even after controlling for clinical covariates. These findings extend prior findings of associations of *DAB2IP* with CAD phenotypes to include survival in those with CAD. Our observed association between rs13007553 (*LINC01250*, intergenic of *MYT1L/TSSC1*) and increased risk of all-cause mortality in patients with CAD is a novel finding among the current literature. These candidate variants do not appear to overlap with the top longevity candidate genes. Additional research is needed to identify genetic contributions to survivorship in those with significant CAD as well as the underlying biological mechanisms of those contributions. Our results will serve as a resource for *in silico* and meta-analyses and can inform the design of future studies. Such work could lead to a better understanding of mortality risk and protective mechanisms in the context of the coronary disease state.”

These corrections have been made to the Results section, subsection Follow-Up Events:

Finally, there were mistakes in the event data summary presented within the text. As above, the White CAD cases in the Discovery group should be 684, not 1,099 as published. The mistakes to the event data summaries were: 1,894 days (5.2 years) should be 2,004 days (5.5 years); 4,302 days (11.7 years) should be 3,953 days (10.8 years); 216 individuals (19.7%) should be 159 individuals (23.3%); 3,304 days (9 years) should be 3,326 days (9.1 years).

“In the discovery dataset (*n* = 684), the median follow-up time was 2,004 days (5.5 years) and maximum follow-up was 3,953 days (10.8 years). At the time of analysis, 159 individuals (23.3%) were deceased on follow-up. In the replication dataset (*n* = 404), the median follow-up was 3,326 days (9.1 years) and the maximum follow-up was 4,420 days (12 years). At the time of analysis, 120 individuals (29.7%) from the replication cohort were deceased on follow-up.”

The authors apologize for this error and state that this does not change the scientific conclusions of the article in any way. The original article has been updated.

## Publisher's Note

All claims expressed in this article are solely those of the authors and do not necessarily represent those of their affiliated organizations, or those of the publisher, the editors and the reviewers. Any product that may be evaluated in this article, or claim that may be made by its manufacturer, is not guaranteed or endorsed by the publisher.
