# Supplementary figures and images for: Genome-Wide Variants Associated With Longitudinal Survival Outcomes Among Individuals With Coronary Artery Disease
Source: Front Genet. 2021 Jun 1;12:661497. doi: 10.3389/fgene.2021.661497 (PMC8204081; doi:10.3389/fgene.2021.661497)

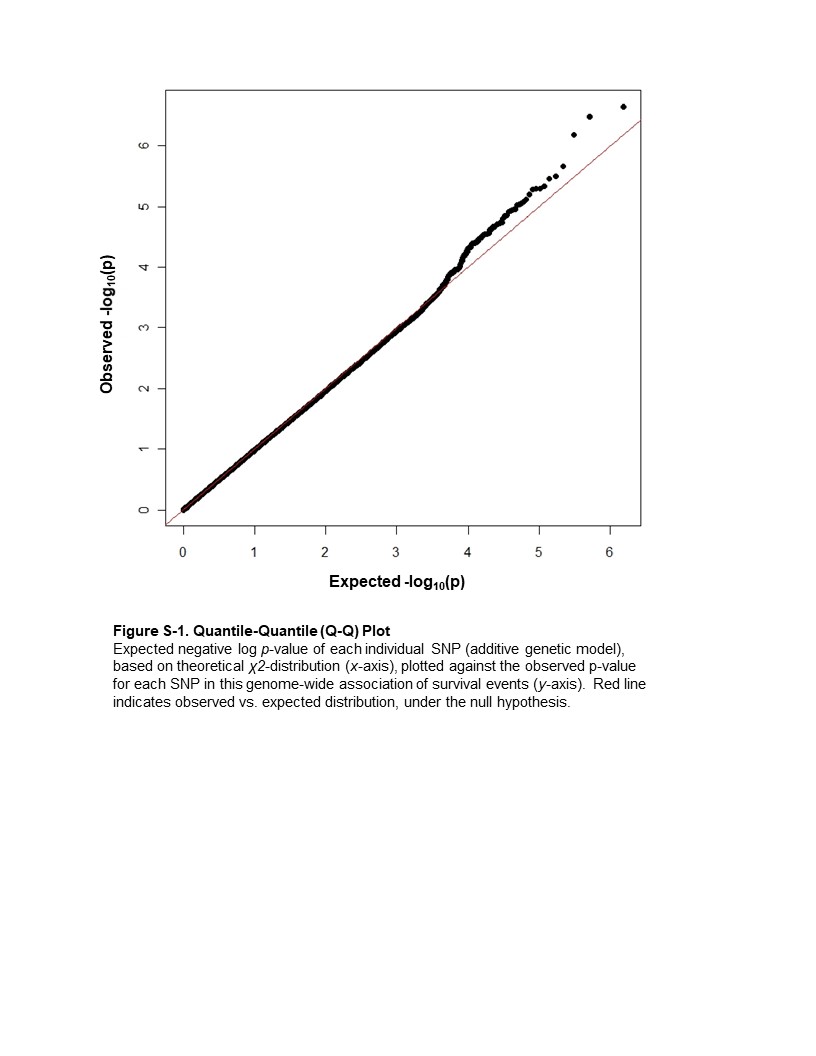

Supplement: Supplementary Figure 1 — Discovery phase Q-Q plot. [file Image_1.JPEG]

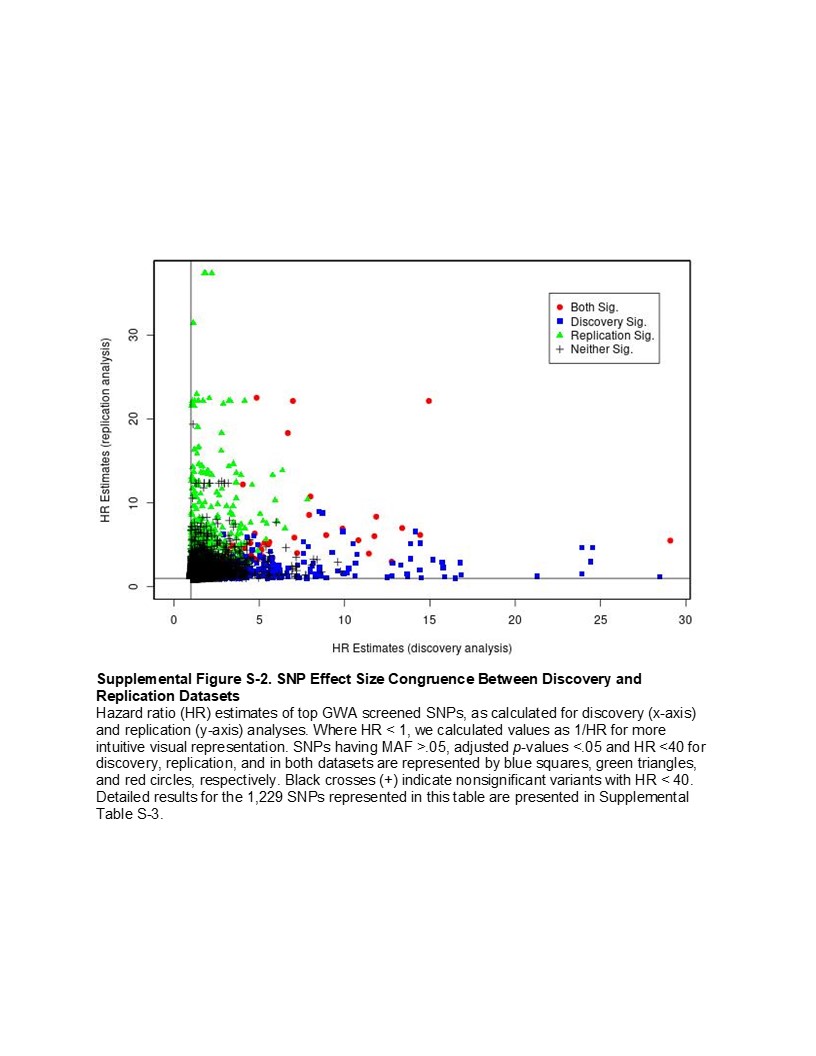

Supplement: Supplementary Figure 2 — SNP effect size congruence between discovery and replication datasets. [file Image_2.JPEG]
