# Supplementary material for: Genome-Wide Variants Associated With Longitudinal Survival Outcomes Among Individuals With Coronary Artery Disease
Source: Front Genet. 2021 Jun 1;12:661497. doi: 10.3389/fgene.2021.661497 (PMC8204081; doi:10.3389/fgene.2021.661497)
Supplement: Supplementary Table 1 — Base model hazard of all-cause mortality among cad cases for discovery-screened SNPs. Less frequent (minor) allele bolded. Bold numerals indicate p < 0.05. ∗Results for base statistical model controlling for age, sex, and four principal components of ancestry. SNP, single nucleotide polymorphism; Chr, chromosome; loc, genomic location (Grch38.p12); MAF, minor allele frequency; HR, hazard ratio; CI, confidence interval; UTR, untranslated region; Kb, kilobase. [file Table_1.docx]

**Supplemental Table 1. Base Model Hazard of All-Cause Mortality Among CAD Cases for Discovery-Screened SNPs**

|  |  |  |  |  |  | *p*-value* | | |  |  |  |
| --- | --- | --- | --- | --- | --- | --- | --- | --- | --- | --- | --- |
| SNP | Allelic Variation | Chr:loc | Variant | MAF | Gene | Discovery | Replication | Meta-analysis | HR | 95% CI | *Z*-score |
| Variants with Negative Direction of Effect (HR < 1) | | | | | | | | | | | |
| rs587936 | T>**A**,***C***,**G** | 9:121582553 | intronic | .38 | *DAB2IP* | **3.75x10-5** | .10 | **2.13x10-5** | .61 | .48-.77 | -4.25 |
| rs118207 | C>**T** | 2:33112067 | intronic | .42 | *LTBP1* | **7.76x10-5** | .57 | **.0005** | .63 | .50-.79 | -3.48 |
| rs9531515 | T>**A**,**G** | 13:31380652 | intergenic | .43 | *B3GALTL/RXFP2* | **4.69x10-5** | .52 | **.0003** | .60 | .47-.77 | -3.59 |
| rs31269 | G>**A**,**C** | 5:76330127 | intronic | .50 | *SV2C* | **3.48x10-5** | .61 | **.0003** | .62 | .49-.78 | -3.59 |
| rs349443 | G>**T** | 1:41811725 | intronic | .38 | *HIVEP3* | **3.67x10-5** | .84 | **.0006** | .58 | .45-.75 | -3.42 |
| rs242413 | C>**T** | 14:56161083 | intronic | .31 | *PELI2* | **1.59x10-5** | .77 | **.0004** | .55 | .42-.72 | -3.52 |
| rs7069959 | **T**>C,G | 10:130871742 | intergenic | .27 | *GLRX3/MIR378C* | **2.93x10-5** | .10 | **.0009** | .55 | .41-.73 | -3.34 |
| Variants with Positive Direction of Effect (HR > 1) | | | | | | | | | | | |
| rs13007553 | **T**>C | 2:3058976 | intronic | .36 | *LINC01250* | **5.93x10-5** | **.04** | **1.05x10-5** | 1.59 | 1.27-1.99 | 4.41 |
| rs13022539 | **A**>G,T | 2:3058283 | intronic | .49 | *LINC01250* | **6.03x10-5** | .24 | **.0001** | 1.59 | 1.27-2.00 | 3.86 |
| rs2108258 | A>**G** | 7:20752375 | intronic | .08 | *ABCB5* | **2.45x10-5** | .05 | **4.98x10-5** | 1.95 | 1.43-2.65 | -4.57 |
| rs17164717 | A>**G** | 7:11527743 | intronic | .02 | *THSD7A* | **1.23x10-5** | .22 | **2.68x10-5** | 3.65 | 2.04-6.53 | -4.12 |
| rs10240390 | A>**C**,**G** | 7:11527648 | intronic | .02 | *THSD7A* | **1.40x10-5** | .28 | **4.49x10-5** | 3.64 | 2.03-6.51 | -4.08 |
| rs1865093 | C>**T** | 19:39445494 | 2Kb upstream | .17 | *SUPT5H* | **5.56x10-5** | .36 | **.0002** | 1.75 | 1.33-2.29 | 3.76 |
| rs7037490 | **A**>C,G | 9:38085787 | intergenic | .16 | *SHB/ALDH1B1* | **3.94x10-5** | .39 | **.0002** | 1.73 | 1.33-2.25 | 3.79 |
| rs7305964 | **G**>A | 12:97499951 | intronic | .32 | *RMST* | **1.97x10-5** | .60 | **.0002** | 1.67 | 1.32-2.11 | -3.68 |
| rs17009399 | G>**C** | 2:73829186 | 5-primeUTR | .08 | *STAMBP* | **3.57x10-5** | .21 | **5.70x10-5** | 2.04 | 1.45-2.86 | -4.03 |
| rs744680 | G>**A** | 10:129943431 | intronic | .20 | *EBF3* | **3.00x10-5** | .61 | **.0003** | 1.71 | 1.33-2.19 | 3.64 |
| rs12620516 | G>**C**,**T** | 2:216553597 | intergenic | .29 | *RPL37A/IGFBP2* | **8.48x10-5** | .63 | **.0008** | 1.58 | 1.26-1.98 | 3.37 |
| rs1865090 | T>**A**,**C** | 19:39441383 | intergenic | .17 | *RPS16/SUPT5H* | **6.70x10-5** | .61 | **.0005** | 1.74 | 1.33-2.28 | -3.48 |
| rs4802033 | G>**A** | 19:39489326 | intergenic | .17 | *TIMM50* | **4.81x10-5** | .63 | **.0004** | 1.76 | 1.34-2.30 | 3.52 |
| rs7305831 | T>**C** | 12:97490908 | intronic | .46 | *RMST* | **3.33x10-7** | .56 | **1.24x10-5** | 1.84 | 1.46-2.32 | 4.37 |
| rs7138358 | **C**>T | 12:97491385 | intronic | .46 | *RMST* | **2.25x10-7** | .58 | **1.04x10-5** | 1.86 | 1.47-2.35 | -4.41 |
| rs10819587 | G>**A** | 9:99019019 | intronic | .11 | *COL15A1* | **2.32x10-6** | .37 | **.0001** | 1.94 | 1.43-2.64 | 3.39 |
| rs4150403 | C>**T** | 2:127292492 | intronic | .09 | *ERCC3* | **4.99x10-5** | .49 | **.0002** | 2.01 | 1.44-2.82 | 3.69 |
| rs2297603 | G>**A**,**T** | 9:99015983 | exonic-missense | .11 | *COL15A1* | **3.37x10-5** | .37 | **.0001** | 1.91 | 1.41-2.60 | 3.82 |
| rs12579455 | A>**G**,**T** | 12:97481554 | intronic | .46 | *RMST* | **6.57x10-7** | .71 | **3.46x10-5** | 1.81 | 1.43-2.28 | 4.14 |
| rs12515837 | T>**A,C** | 5:30940209 | intergenic | .05 | *LOC729862/CDH6* | **4.08x10-5** | .52 | **.0003** | 2.41 | 1.58-3.66 | -3.66 |
| rs17009433 | T>**C** | 2:73872884 | intronic | .10 | *STAMBP* | **3.16x10-6** | .84 | **.0001** | 2.08 | 1.53-2.83 | -3.81 |
| rs896651 | **T**>A,G | 18: 61564558 | intergenic | .35 | *CDH20/RNF152* | **6.07x10-5** | .94 | **.001** | 1.62 | 1.28-2.04 | 3.19 |
| rs11126419 | C>**T** | 2:73864992 | intronic | .09 | *STAMBP* | **1.84x10-5** | .59 | **.0002** | 2.06 | 1.48-2.86 | 3.78 |
| Less frequent (minor) allele bolded. Bold numerals indicate *p* < 0.05. *Results for base statistical model controlling for age, sex, and four principal components of ancestry. SNP, single nucleotide polymorphism; Chr, chromosome; loc, genomic location (GRCh38.p12); MAF, minor allele frequency; HR, hazard ratio; CI, confidence interval; UTR, untranslated region; Kb, kilobase | | | | | | | | | | | |
