# Supplementary material for: Genome-Wide Variants Associated With Longitudinal Survival Outcomes Among Individuals With Coronary Artery Disease
Source: Front Genet. 2021 Jun 1;12:661497. doi: 10.3389/fgene.2021.661497 (PMC8204081; doi:10.3389/fgene.2021.661497)
Supplement: Supplementary Table 2 — Top 93 discovery SNPs. ∗Base model adjusting for age, sex, and four principal components of ancestry; p(adj), clinically adjusted model controlling for body mass index (BMI), history of smoking, type 2 diabetes, hyperlipidemia, hypertension, creatinine, and ejection fraction. Bold indicates GWAS-level significance p = 10–8. [file Table_2.docx]

**Supplemental Table S-2. Top 93 Discovery SNPs**

| **SNP** | **Gene** | **Chr** | **MAF** | ***P**** | ***P* (adj)** | **HR** | **95% CI** | | | |
| --- | --- | --- | --- | --- | --- | --- | --- | --- | --- | --- |
|  |  |  |  |  |  |  | **Upper Lower** | | | |
| rs7138358 | *MIR1251,RMST* | 12 | .46 | 2.25x10-7 | **5.03x10-8** | 1.86 | | 1.47 | 2.35 |  |
| rs7305831 | *MIR1251,RMST* | 12 | .45 | 3.33x10-7 | **4.34x10-8** | 1.84 | | 1.46 | 2.32 |  |
| rs12579455 | *RMST* | 12 | .46 | 6.57x10-7 | **7.92x10-8** | 1.81 | | 1.43 | 2.28 |  |
| rs17103766 | *BRMS1L/MBIP* | 14 | .04 | 2.18x10-6 | **5.03x10-8** | 2.81 | | 1.83 | 4.31 |  |
| rs17009433 | *STAMBP* | 2 | .10 | 3.16x10-6 | 9.15x10-5 | 2.08 | | 1.53 | 2.83 |  |
| rs743980 | *RPS6KA2* | 6 | .16 | 3.46x10-6 | 1.70x10-5 | 1.92 | | 1.46 | 2.53 |  |
| rs4722966 | *FKBP14* | 7 | .07 | 5.25x10-6 | 1.97x10-5 | 2.22 | | 1.58 | 3.14 |  |
| rs11970312 | *RPS6KA2* | 6 | .16 | 7.69x10-6 | 7.46x10-5 | 1.86 | | 1.42 | 2.44 |  |
| rs1450397 | *FSTL5/NAF1* | 4 | .18 | 8.28x10-6 | 1.10x10-4 | 1.81 | | 1.40 | 2.36 |  |
| rs12099933 | *HOXC11* | 12 | .02 | 8.93x10-6 | 3.37x10-6 | 3.34 | | 1.96 | 5.68 |  |
| rs4776247 | *UNC13C/RSL24D1* | 15 | .09 | 9.14x10-6 | 9.94x10-5 | 2.09 | | 1.51 | 2.89 |  |
| rs12150051 | *FLJ34690* | 17 | .42 | 1.12x10-5 | 5.44x10-5 | 0.58 | | .46 | .74 |  |
| rs17009428 | *STAMBP* | 2 | .12 | 1.12x10-5 | 1.03x10-4 | 1.92 | | 1.43 | 2.56 |  |
| rs3734114 | *ATG10* | 5 | .19 | 1.16x10-5 | 4.76x10-6 | 1.73 | | 1.35 | 2.20 |  |
| rs9932462 | *EMP2/TEKT5* | 16 | .01 | 1.17x10-5 | 1.47x10-3 | 4.28 | | 2.23 | 8.19 |  |
| rs17164717 | *THSD7A* | 7 | .02 | 1.23x10-5 | 1.77x10-6 | 3.65 | | 2.04 | 6.53 |  |
| rs10240390 | *THSD7A* | 7 | .02 | 1.40x10-5 | 2.27x10-6 | 3.64 | | 2.03 | 6.51 |  |
| rs1118864 | *FSTL5/NAF1* | 4 | .18 | 1.41x10-5 | 1.66x10-4 | 1.79 | | 1.38 | 2.33 |  |
| rs10519327 | *STAMBP/ACTG2* | 2 | .13 | 1.43x10-5 | 1.98x10-4 | 1.90 | | 1.42 | 2.55 |  |
| rs242413 | *PELI2* | 14 | .30 | 1.59x10-5 | 7.67x10-7 | 0.55 | | .42 | .72 |  |
| rs11126419 | *STAMBP* | 2 | .09 | 1.84x10-5 | 4.29x10-5 | 2.06 | | 1.48 | 2.86 |  |
| rs2148707 | *MRP63/MIPEPP3* | 13 | .24 | 1.85x10-5 | 1.87x10-5 | 1.69 | | 1.33 | 2.15 |  |
| rs6924732 | *RPS6KA2* | 6 | .17 | 1.88x10-5 | 8.62x10-5 | 1.82 | | 1.38 | 2.39 |  |
| rs17321135 | *CNTNAP5* | 2 | .42 | 1.95x10-5 | 3.12x10-6 | 1.63 | | 1.30 | 2.04 |  |
| rs7305964 | *RMST* | 12 | .32 | 1.97x10-5 | 1.00x10-5 | 1.67 | | 1.32 | 2.11 |  |
| rs17623915 | *AKAP13* | 15 | .22 | 2.05x10-5 | 1.63x10-5 | 1.67 | | 1.32 | 2.12 |  |
| rs10904623 | *LOC439949/LOC100507127* | 10 | .23 | 2.08x10-5 | 1.30x10-5 | 1.68 | | 1.32 | 2.13 |  |
| rs2062640 | *UNC13C/RSL24D1* | 15 | .11 | 2.15x10-5 | 3.20x10-5 | 1.97 | | 1.44 | 2.70 |  |
| rs12761476 | *LOC439949/LOC100507127* | 10 | .23 | 2.17x10-5 | 1.56x10-6 | 1.67 | | 1.32 | 2.12 |  |
| rs6857753 | *FSTL5/NAF1* | 4 | .20 | 2.31x10-5 | 4.72x10-4 | 1.78 | | 1.36 | 2.33 |  |
| rs10819587 | *COL15A1* | 9 | .11 | 2.32x10-5 | 1.82x10-4 | 1.94 | | 1.43 | 2.64 |  |
| rs10030251 | *FSTL5/NAF1* | 4 | .20 | 2.42x10-5 | 2.92x10-4 | 1.77 | | 1.36 | 2.30 |  |
| rs2108258 | *ABCB5* | 7 | .08 | 2.45x10-5 | 1.11x10-4 | 1.95 | | 1.43 | 2.65 |  |
| rs12508239 | *FSTL5/NAF1* | 4 | .26 | 2.78x10-5 | 4.51x10-4 | 1.63 | | 1.30 | 2.05 |  |
| rs1868695 | *FSTL5/NAF1* | 4 | .20 | 2.79x10-5 | 3.47x10-4 | 1.76 | | 1.35 | 2.29 |  |
| rs238753 | *NHLRC2/ADRB1* | 10 | .04 | 2.82x10-5 | 4.02x10-4 | 2.48 | | 1.62 | 3.80 |  |
| rs11100437 | *FSTL5/NAF1* | 4 | .20 | 2.83x10-5 | 3.58x10-4 | 1.76 | | 1.35 | 2.29 |  |
| rs4961018 | *CNBD1* | 8 | .28 | 2.87x10-5 | 1.62x10-5 | .55 | | .42 | .73 |  |
| rs2905501 | *CD6/CD5* | 11 | .08 | 2.88x10-5 | 3.02x10-4 | 1.97 | | 1.43 | 2.70 |  |
| rs7069959 | *GLRX3/MIR378C* | 10 | .27 | 2.93x10-5 | 1.85x10-4 | .55 | | .41 | .73 |  |
| rs13138079 | *FSTL5/NAF1* | 4 | .21 | 2.98x10-5 | 2.63x10-4 | 1.75 | | 1.35 | 2.27 |  |
| rs744680 | *EBF3* | 10 | .20 | 3.00x10-5 | 2.78x10-5 | 1.71 | | 1.33 | 2.19 |  |
| rs7902859 | *NHLRC2/ADRB1* | 10 | .04 | 3.08x10-5 | 4.48x10-4 | 2.51 | | 1.63 | 3.86 |  |
| rs238749 | *NHLRC2/ADRB1* | 10 | .04 | 3.20x10-5 | 4.72x10-4 | 2.50 | | 1.62 | 3.85 |  |
| rs6770134 | *LSAMP-AS3/IGSF11* | 3 | .31 | 3.24x10-5 | 1.05x10-5 | 1.62 | | 1.29 | 2.04 |  |
| rs2297603 | *COL15A1* | 9 | .11 | 3.37x10-5 | 2.49x10-4 | 1.91 | | 1.41 | 2.60 |  |
| rs2592980 | *SETD7* | 4 | .17 | 3.39x10-5 | 2.22x10-4 | 1.79 | | 1.36 | 2.36 |  |
| rs31269 | *SV2C* | 5 | .50 | 3.48x10-5 | 2.35x10-5 | .62 | | .49 | .77 |  |
| rs17009399 | *STAMBP* | 2 | .08 | 3.57x10-5 | 9.30x10-5 | 2.04 | | 1.45 | 2.86 |  |
| rs349443 | *HIVEP3* | 1 | .37 | 3.67x10-5 | 7.19x10-5 | .58 | | .45 | .75 |  |
| rs587936 | *DAB2IP* | 9 | .38 | 3.75x10-5 | 4.79x10-4 | .61 | | .48 | .77 |  |
| rs1556060 | *DCLK1* | 13 | .12 | 3.81x10-5 | 1.63x10-5 | 1.89 | | 1.40 | 2.57 |  |
| rs6939941 | *RPS6KA2* | 6 | .25 | 3.88x10-5 | 7.42x10-5 | 1.67 | | 1.31 | 2.12 |  |
| rs1410641 | *DCLK1/SOHLH2* | 13 | .12 | 3.94x10-5 | 2.15x10-5 | 1.89 | | 1.39 | 2.56 |  |
| rs7037490 | *SHB/ALDH1B1* | 9 | .16 | 3.94x10-5 | 1.07x10-3 | 1.73 | | 1.33 | 2.25 |  |
| rs4688076 | *LSAMP-AS3/IGSF11* | 3 | .31 | 4.00x10-5 | 9.60x10-6 | 1.61 | | 1.28 | 2.03 |  |
| rs1993024 | *FSTL5/NAF1* | 4 | .26 | 4.04x10-5 | 6.42x10-4 | 1.61 | | 1.28 | 2.03 |  |
| rs11727755 | *FSTL5/NAF1* | 4 | .20 | 4.07x10-5 | 4.84x10-4 | 1.73 | | 1.33 | 2.24 |  |
| rs921343 | *FSTL5/NAF1* | 4 | .20 | 4.07x10-5 | 4.84x10-4 | 1.73 | | 1.33 | 2.24 |  |
| rs12515837 | *LOC729862/CDH6* | 5 | .05 | 4.08x10-5 | 2.75x10-4 | 2.40 | | 1.58 | 3.66 |  |
| rs2835913 | *KCNJ6* | 21 | .03 | 4.14x10-5 | 6.97x10-5 | 2.72 | | 1.69 | 4.39 |  |
| rs17158483 | *SCRN1* | 7 | .08 | 4.22x10-5 | 1.49x10-4 | 2.03 | | 1.45 | 2.85 |  |
| rs4480147 | *EXT1* | 8 | .03 | 4.24x10-5 | 6.12x10-4 | 2.69 | | 1.68 | 4.33 |  |
| rs3016176 | *CD6/CD5* | 11 | .10 | 4.43x10-5 | 4.81x10-4 | 1.90 | | 1.40 | 2.59 |  |
| rs9531515 | *B3GALTL/RXFP2* | 13 | .43 | 4.69x10-5 | 9.43x10-6 | .60 | | .47 | .77 |  |
| rs82625 | *NHLRC2/ADRB1* | 10 | .04 | 4.72x10-5 | 7.13x10-04 | 2.32 | | 1.55 | 3.48 |  |
| rs10489483 | *LOC100288079/HMCN1* | 1 | .13 | 4.74x10-5 | 9.63x10-5 | 1.77 | | 1.34 | 2.33 |  |
| rs7034450 | *GRIN3A/CYLC2* | 9 | .32 | 4.79x10-5 | 9.11x10-4 | 1.64 | | 1.29 | 2.09 |  |
| rs4802033 | *TIMM50* | 19 | .17 | 4.81x10-5 | 3.71x10-4 | 1.76 | | 1.34 | 2.30 |  |
| rs4150403 | *ERCC3* | 2 | .09 | 4.99x10-5 | 5.62x10-5 | 2.01 | | 1.44 | 2.82 |  |
| rs7774664 | *CMAHP/LRRC16A* | 6 | .33 | 5.14x10-5 | 2.05x10-5 | 1.59 | | 1.27 | 1.99 |  |
| rs4688173 | *SYNPR* | 3 | .33 | 5.37x10-5 | 8.24x10-5 | .58 | | .45 | .76 |  |
| rs1865093 | *SUPT5H* | 19 | .17 | 5.56x10-5 | 6.34x10-4 | 1.75 | | 1.33 | 2.29 |  |
| rs9580025 | *MRP63/MIPEPP3* | 13 | .24 | 5.58x10-5 | 6.42x10-5 | 1.64 | | 1.29 | 2.09 |  |
| rs386958 | *BRMS1L/MBIP* | 14 | .06 | 5.69x10-5 | 7.22x10-4 | 2.18 | | 1.49 | 3.19 |  |
| rs13007553 | *LINC01250(MYT1L/TSSC1)* | 2 | .36 | 5.93x10-5 | 1.07x10-3 | 1.59 | | 1.27 | 1.99 |  |
| rs13022539 | *LINC01250(MYT1L/TSSC1)* | 2 | .48 | 6.03x10-5 | 9.15x10-5 | 1.59 | | 1.27 | 2.00 |  |
| rs896651 | *CDH20/RNF152* | 18 | .35 | 6.07x10-5 | 2.46x10-5 | 1.62 | | 1.28 | 2.04 |  |
| rs4712901 | *CMAHP/LRRC16A* | 6 | .35 | 6.19x10-5 | 2.05x10-5 | 1.59 | | 1.27 | 1.99 |  |
| rs1534300 | *LOC401177/CDH18* | 5 | .21 | 6.30x10-5 | 4.84x10-6 | 1.65 | | 1.29 | 2.12 |  |
| rs2278816 | *PLXNA4* | 7 | .22 | 6.68x10-5 | 6.68x10-5 | 1.67 | | 1.30 | 2.15 |  |
| rs1865090 | *SUPT5H* | 19 | .17 | 6.70x10-5 | 7.41x10-4 | 1.74 | | 1.32 | 2.28 |  |
| rs6740655 | *SLC8A1/LOC388942* | 2 | .34 | 6.77x10-5 | 4.17x10-6 | .60 | | .47 | .77 |  |
| rs7000721 | *CNBD1* | 8 | .35 | 7.17x10-5 | 9.24x10-5 | .60 | | .46 | .77 |  |
| rs11253614 | *LOC439949/LOC100507127* | 10 | .23 | 7.70x10-5 | 4.78x10-5 | 1.59 | | 1.26 | 2.01 |  |
| rs118207 | *LTBP1* | 2 | .42 | 7.76x10-5 | 4.43x10-4 | .62 | | .49 | .79 |  |
| rs4731850 | *PLXNA4* | 7 | .22 | 7.91x10-5 | 9.69x10-5 | 1.66 | | 1.29 | 2.14 |  |
| rs6968906 | *PLXNA4* | 7 | .23 | 8.01x10-5 | 6.54x10-5 | 1.65 | | 1.29 | 2.13 |  |
| rs12620516 | *RPL37A/IGFBP2* | 2 | .29 | 8.48x10-5 | 7.40x10-4 | 1.58 | | 1.26 | 1.98 |  |
| rs7674180 | *SORBS2* | 4 | .05 | 8.58x10-5 | 1.35x10-5 | 2.18 | | 1.48 | 3.21 |  |
| rs6741495 | *MARCHF4* | 2 | .05 | 9.11x10-5 | 2.16x10-4 | 2.37 | | 1.54 | 3.64 |  |
| rs7578835 | *CNTNAP5* | 2 | .38 | 9.24x10-5 | 8.97x10-5 | 1.57 | | 1.25 | 1.96 |  |
| rs7833568 | *CNBD1* | 8 | .27 | 9.83x10-5 | 3.94x10-5 | .58 | | .44 | .76 |  |

*Base model adjusting for age, sex, and four principal components of ancestry; *P*(adj), clinically adjusted model controlling for body mass index (BMI), history of smoking, type 2 diabetes, hyperlipidemia, hypertension, creatinine, and ejection fraction. Bold indicates GWAS-level significance p=10^-8^.
